# Preferences for Colorectal Cancer Screening Modalities Among the General Population in Saudi Arabia

**DOI:** 10.7759/cureus.36020

**Published:** 2023-03-11

**Authors:** Mohammad Alharbi, Lena S AlSaleem, Maha H Alrashid, Halal Alutaibi, Shahad A Alabdrabulrida, Ahood A Mahjari, Rahaf AlZahrani

**Affiliations:** 1 Surgery, Imam Mohammad Ibn Saud Islamic University (IMSIU), Riyadh, SAU; 2 Medical Intern, Imam Mohammad Ibn Saud Islamic University (IMSIU), Riyadh, SAU; 3 Medical Intern, King Faisal University, Hofouf, SAU; 4 College of Medicine, King Faisal University, Al-Ahsa, SAU; 5 College of Medicine, Najran University, Najran, SAU

**Keywords:** saudi arabia, cancer, screening, colon, colorectal

## Abstract

Background: Colorectal cancer (CRC) could be a leading explanation for cancer-related death. Numerous studies have shown the benefit of early screening for colorectal cancer in reducing mortality. Screening for colorectal cancer is a rational and cost-effective strategy for reducing the incidence of colorectal cancer and related mortality. Despite endorsement by academic and healthcare organizations, patient awareness and compliance with screening are low, partly due to patient-related barriers to screening.

Aim: This study aimed to explore the preferred screening method for colorectal cancer in Saudi Arabia in general.

Methods: This is a cross-sectional study conducted among the Saudi adult population from September 2021 through February 2022. A self-developed questionnaire was distributed among the population using an online platform. Data were tabulated in Google Forms, and all statistical analyses were performed using SPSS version 26 (IBM Corp., Armonk, NY).

Results: During this study, data from 10,781 participants were analyzed. Among them, the most preferred screening modality for colorectal cancer was the stool fecal immunochemical test (41.7%) and the most suitable (33.5%). The most commonly mentioned qualities that influenced choosing a particular screening test were "how the test was performed" (50.4%).

Conclusion: Because the stool fecal immunochemical test is the most preferred screening modality for colorectal cancer, this study could serve as a database to aid in the implementation of a colorectal cancer screening program that meets the preferences of the general population of Saudi Arabia.

## Introduction

Colorectal cancer (CRC) could be a leading explanation for cancer-related death. Numerous studies have shown the benefit of early screening for colorectal cancer in reducing mortality. However, global screening rates are still low. This study assesses the awareness of CRC risk factors, warning signs, attitudes towards CRC guidelines and screening modalities, and perceived benefits of the screening to identify the most preferable CRC screening test [1]. In addition, this study will investigate screening intentions and previous uptake of CRC screening tests in the general population in Saudi Arabia [2]. Screening for colorectal cancer could be a rational and cost-effective strategy for reducing the incidence of colorectal cancer and related mortality. Despite endorsement by academic and healthcare organizations, patient awareness and compliance with screening are low, partly thanks to patient-related barriers to screening [3]. Early detection of CRC is recommended due to the increased risk of postoperative sepsis after surgery for colon cancer in individuals over 65 who have a BMI ≥ 30 kg/m2, an ASA score of 2 or higher, and additional comorbidities such as diabetes and cardiovascular disease [4,5]. So, it is crucial to understand preferences for screening within the Saudi population, where relatively few people have had CRC screening. A key factor driving the success or failure of any screening program is patients' willingness to undergo the screening test [6]. People in Saudi Arabia should be more aware of colorectal cancer incidence and prognosis, as well as willing to undergo screening with different tests [7]. Only if evidence-based initiatives like the mailed fecal immunochemical test (FIT) outreach are put into practice will they have an impact on health outcomes. However, efforts to put programs in place are frequently constrained by organizational-level issues [8]. There are several colorectal cancer screening procedures available, each with a distinct level of accuracy, suggested frequency, and administration. These tests include annual fecal occult blood testing (FOBT), flexible sigmoidoscopy (FSIG), every five years, and both annual FOBT and FSIG. According to a review from the USA, the five screening methods listed below were all acceptable and fairly equally cost-effective for "average-risk" people starting at age 50: fecal occult blood testing every year, flexible sigmoidoscopy every five years, a combination of fecal occult blood testing every year and flexible sigmoidoscopy every five years, colonoscopy every ten years, and double-contrast barium enema every five to ten years [9]. Based on the research on screening for colorectal cancer in the USA, a comparison was done on a set of personal traits that link with preferences for colorectal cancer screening test qualities, previous colorectal cancer screening behavior, and future colorectal cancer screening intentions [10]. Recommendations for colorectal cancer screening encourage patients to decide on various screening methods that support individual preferences for benefits, risks, screening frequency, and discomfort. A model was devised as an instance of how individuals with varying tolerance for screening complications risk might choose their preferred screening strategy [11-12]. In order to evaluate patients' preferences for mCRC treatment and the relative importance of cost, efficacy improvement, side effect avoidance, and therapeutic convenience, a study was conducted in Singapore to examine patient preferences and the anticipated relative uptake for targeted therapies in mCRC [13]. The adoption of colonoscopy as the primary method for screening for colorectal cancer (CRC) without evidence of patient preferences has been discussed in several studies. An investigation into patients' preferences for the National Cancer Screening Program's (NCSP) CRC primary screening test was carried out in Korea [14]. Furthermore, the majority of the previous studies in Saudi Arabia recruited visitors to shopping malls who were not representative of the general population to assess the knowledge and determinants of CRC screening among the population in Jeddah, Saudi Arabia [15]. A significant influence is generated on the patient-physician interaction by primary care physicians (PCPs), who play a crucial role in providing colorectal cancer (CRC) screening. For instance, colorectal cancer was the second most common cancer in men and the third in women in Thailand. Early screening and surveillance can reduce colorectal cancer morbidity and mortality, and the number of patients with colorectal cancer of all genders has been rapidly increasing. The standard screening guideline for colorectal cancer recommended by national expert groups is to start in asymptomatic, average-risk adults at the age of fifty by the primary care physicians who have a role in arranging and referring patients for colorectal cancer screening. Colonoscopy is the most preferred screening tool [16]. Patients with limited literacy skills are less likely to be knowledgeable about CRC screening compared to patients with adequate literacy skills. A study was conducted in the USA about the effect of health literacy on knowledge of and receipt of colorectal cancer screening. An estimated half of Americans have limited health literacy skills. Low literacy has been associated with less receipt of preventive services. So, primary care providers should ensure patients' understanding of CRC screening when discussing screening options [17-18]. The discussion of patient values and preferences is a critical step in engaging patients to participate in medical decision-making [9]. In conclusion, the public of Saudi Arabia has become more aware of the growing threat of colorectal cancer (CRC) because it is widely thought to be a deadly disease with life-threatening side effects from chemotherapy. It is interesting to note that diagnosis at an early age is noted in both old and new epidemiological reports. The right side of the colon is increasingly recognized as an unusual presentation of CRC. Saudi cancer registry data revealed that males and females in the kingdom experience CRC at a rate of 51% and 62%, respectively, compared to worldwide rates, with 65% and 77% mortality rates reported, respectively, in 1993-2003. All genders are vulnerable to CRC. Despite that, Saudi females have the highest incidence and mortality rates when compared with other populations in less developed areas. Saudi Arabia has public and private tertiary healthcare centers that offer colonoscopies in major cities. But, in the absence of an organized screening program on a national level in Saudi Arabia, colonoscopy is not accessible for screening purposes. Additionally, performing a screening colonoscopy privately would be prohibitively expensive for an average-income individual. There is no actual explanation for the barriers to early detection or organized screening yet. Our study intended to explore the preferable screening tool for CRC. We hypothesize that FIT would be the most preferred CRC screening test among the general population of Saudi Arabia.

## Materials and methods

The population of Saudi Arabia was studied in this descriptive cross-sectional study. The study was carried out in Saudi Arabia between September 2021 and February 2022. A self-administered software questionnaire was distributed to the general public at random via social media. The mid-year population in Saudi Arabia in 2020 was used for sample size calculation, and the values were placed in the level of precision formula with a margin error determined as 5% and a confidence level of 95% that yielded a sample size of 385 [19]. The total number of participants in this study was 10,781. Before the online survey began, all participants were given a webpage describing the study's goals and asked for informed consent. Every survey response was connected to an internet protocol in order to prevent responses from being repeated. The Medical College Institutional Review Board at Al-Imam Mohammad Ibn Saud Islamic University, Riyadh, Saudi Arabia, approved this study protocol (project number: 99-2021).

The general population in Saudi Arabia of either gender, aged 18 years and above, who were able to read and write Arabic (the official language of Saudi Arabia) were included in the study. Adults outside of Saudi Arabia and those less than 18 years old or diagnosed with colorectal cancer were excluded from this study.

A self-developed questionnaire included 22 questions: 13 asked about socio-demographic characteristics, and the other 9 were about the preferences of the general population of Saudi Arabia regarding colorectal screening methods. Two consultants validated this questionnaire, and a pilot study was performed on 20 people prior to distribution.

The data were analyzed using Statistical Packages for Social Sciences (SPSS) version 26 (IBM Corp., Armonk, NY, USA). Both descriptive and inferential statistics were conducted. In descriptive statistics, all categorical variables were presented as numbers and percentages. The relationship between the colorectal cancer screening preference and the socio-demographic characteristics of participants has been examined using the Chi-square test. A p-value cutoff point of 0.05 at 95% CI was used to determine statistical significance.

## Results

In total, 10,781 participants were involved. Table 1 presented the socio-demographic characteristics of the participants. The most common age group was 18-25 years old (55.9%), with females dominating the males (67% vs. 33%). Respondents living in the western region constitute 24.5%, while those living in the northern region constitute 22.8%. Most respondents were of Saudi nationality (96%) and nearly two-thirds had bachelor’s degrees (64.1%). The proportion of participants who were working in the medical field was 3.8%. Furthermore, the majority of the respondents were unemployed (62.5%), and 63.7% had a monthly income of less than 6,000 SAR per month. The prevalence of participants who had an associated chronic disease was 17.4%. Additionally, a family history of colorectal cancer was found among 4.7%.

**Table 1:** Socio-demographic characteristics of participants (n=10781)

Study variables	N (%)
Age group	
18–25 years	6029 (55.9%)
26–40 years	2849 (26.4%)
41–50 years	1289 (12.0%)
51–60 years	486 (04.5%)
61–70 years	104 (01.0%)
>70 years	24 (0.20%)
Gender	
Male	3559 (33.0%)
Female	7222 (67.0%)
Residence region	
Eastern region	1896 (17.6%)
Western region	2643 (24.5%)
Northern region	2462 (22.8%)
Southern region	2180 (20.2%)
Central Region	1600 (14.8%)
Nationality	
Saudi	10349 (96.0%)
Non-Saudi	432 (04.0%)
Educational level	
Primary	225 (02.1%)
Secondary	3222 (29.9%)
Bachelor’s degree	6908 (64.1%)
Postgraduate studies	426 (04.0%)
Working in the medical field	
Yes	406 (03.8%)
No	10375 (96.2%)
Employment status	
Employed full time	3060 (28.4%)
Employed part time	478 (04.4%)
Self-employed	505 (04.7%)
Unemployed	6738 (62.5%)
Monthly income (SAR)	
<6000	6866 (63.7%)
6001 – 12,000	2224 (20.6%)
12,001 – 25,000	1415 (13.1%)
>25,000	276 (02.6%)
Associated chronic disease	
Yes	1877 (17.4%)
No	8904 (82.6%)
Family history of cancer	
Colorectal cancer	504 (04.7%)
Other	561 (05.2%)
No cancer/do not know	9716 (90.1%)

**Figure 1:**
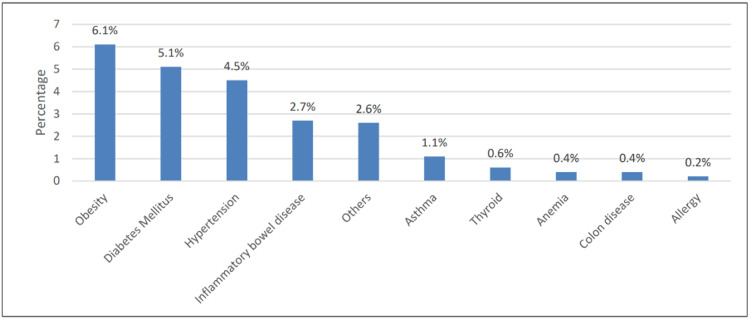
Specific type of chronic disease

In Figure 1, the most commonly associated chronic disease was obesity (6.1%), followed by diabetes (5.1%) and hypertension (4.5%). 

**Figure 2:**
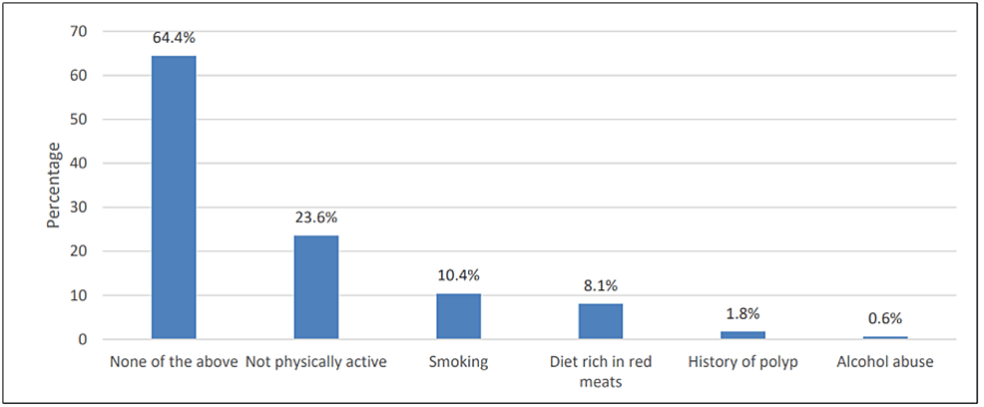
Knowledge about the risk factor of colorectal cancer

In Figure 2, respondents believed that the most common risk factors for colorectal cancer were physical inactivity (23.6%) and smoking (10.4%).

**Figure 3:**
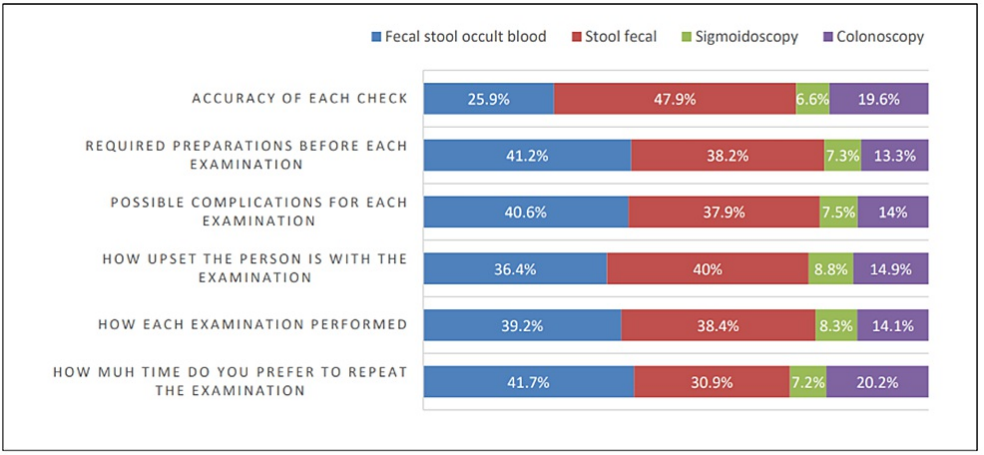
Preferred screening test modality according to a pattern of examination

In Figure 3, respondents preferred the stool fecal immunochemical test because it was more accurate (47.9%) and because they had problems with other screening methods (40%). Other participants preferred fecal stool occult blood in terms of screening interval (41.7%), preparation required prior to screening (41.2%), complications from other screening modalities (40.6%), and screening method (39.2%).

Participants' behavior regarding colorectal screening was given in Table 2. It can be observed that most of the respondents did not discuss the early colorectal cancer screening methods with their doctors (92.8%), and most of them had not tried the screening test (93.4%), with only 2.3% of the respondents having tried the fecal stool occult blood test. The most commonly mentioned qualities that influenced choosing a particular screening test were "how the test was performed" (50.4%) and the accuracy of the test (46.9%). Stool fecal immunochemical testing was the most common (33.5%) and preferable (41.7%) screening modality in the study population.

**Table 2:** Participants’ behavior regarding colorectal cancer screening (n=10781)

Variables	N (%)
Have you ever discussed with your doctor about early screening for colorectal cancer?	
Yes	299 (02.8%)
No	10001 (92.8%)
I do not remember	481 (04.5%)
Have you had any of the following colon and rectal screening before?*	
No, I have not	10067 (93.4%)
Fecal stool occult blood; stool samples provided in a container to check for blood	251 (02.3%)
Stool fecal immunochemical test; stool sample taken by a brush to check the presence of blood by antibodies	88 (0.80%)
Sigmoidoscopy; flexible tube inserted up to the sigmoid colon	120 (01.1%)
Colonoscopy; flexible tube is inserted which examines the large bowel and distal part of the small bowel	163 (01.5%)
Colorectal consultation	334 (03.1%)
Most important qualities of a test that influenced your decision on choosing a particular test*	
How the test is performed	5434 (50.4%)
Accuracy of the test	5057 (46.9%)
Frequency of the test	4931 (45.7%)
Complications of the test	4086 (37.9%)
Discomfort from the test	4066 (37.7%)
Preparation for the test	2052 (19.0%)
What to do if the test is abnormal	1643 (15.2%)
Most suitable screening modality	
Fecal stool occult blood	3207 (29.7%)
Stool fecal immunochemical test	3613 (33.5%)
Sigmoidoscopy	800 (07.4%)
Colonoscopy	3161 (29.3%)
Most preferred screening modality	
Fecal stool occult blood	3339 (31.0%)
Stool fecal immunochemical test	4498 (41.7%)
Sigmoidoscopy	772 (07.2%)
Colonoscopy	2172 (20.1%)

When measuring the relationship between the most preferred screening modality and the demographic characteristics of the participants, we found that the prevalence of respondents who preferred the stool fecal immunochemical test was more common among those who were 18-25 years old (p < 0.001), females (p < 0.001), those living in the Western region (p < 0.001), unemployed participants (p < 0.001), those who earned less than 6,000 SAR per month (p < 0.001), and those with a family history of cancer (p = 0.001), while the prevalence of respondents who preferred colonoscopy was more common among those who have associated chronic diseases (p = 0.003) (Table 3).

**Table 3:** Relationship between the most preferred colorectal screening modality and the socio-demographic characteristics of participants (n=10781)

Study variables	Most preferred colorectal cancer screening modality				P-value^§^
	Fecal occult N (%)^(n=3339)^	Stool fecal N (%)^(n=4498)^	Sigmoidoscopy N (%)^(n=772)^	Colonoscopy N (%)^(n=2172)^	
Age group					
18–25 years	1660 (49.7%)	2835 (63.0%)	453 (58.7%)	1081 (49.8%)	<0.001**
26–40 years	974 (29.2%)	1078 (24.0%)	183 (23.7%)	614 (28.3%)	
41–50 years	455 (13.6%)	384 (08.5%)	99 (12.8%)	351 (16.2%)	
>50 years	250 (07.5%)	201 (04.5%)	37 (04.8%)	126 (05.8%)	
Gender					
Male	1172 (35.1%)	1369 (30.4%)	279 (36.1%)	739 (34.0%)	<0.001**
Female	2167 (64.9%)	3129 (69.6%)	493 (63.9%)	1433 (66.0%)	
Residence region					
Eastern region	597 (17.9%)	834 (18.5%)	120 (15.5%)	345 (15.9%)	<0.001**
Western region	757 (22.7%)	1203 (26.7%)	179 (23.2%)	504 (23.2%)	
Northern region	927 (27.8%)	797 (17.7%)	196 (25.4%)	542 (25.0%)	
Southern region	645 (19.3%)	854 (19.0%)	179 (23.2%)	502 (23.1%)	
Central region	413 (12.4%)	810 (18.0%)	98 (12.7%)	279 (12.8%)	
Nationality					
Saudi	3212 (96.2%)	4328 (96.2%)	733 (94.9%)	2076 (95.6%)	0.249
Non-Saudi	127 (03.8%)	170 (03.8%)	39 (05.1%)	96 (04.4%)	
Educational level					
Secondary or below	1072 (32.1%)	1454 (32.3%)	254 (32.9%)	667 (30.7%)	0.535
Bachelor’s degree or higher	2267 (67.9%)	3044 (67.7%)	518 (67.1%)	1505 (69.3%)	
Working in the medical field					
Yes	107 (03.2%)	177 (03.9%)	32 (04.1%)	90 (04.1%)	0.220
No	3232 (96.8%)	4321 (96.1%)	740 (95.9%)	2082 (95.9%)	
Employment status					
Employed	1378 (41.3%)	1485 (33.0%)	291 (37.7%)	889 (40.9%)	<0.001**
Unemployed	1961 (58.7%)	3013 (67.0%)	481 (62.3%)	1283 (59.1%)	
Monthly income (SAR)					
<6000	2023 (60.6%)	3045 (67.7%)	496 (64.2%)	1302 (59.9%)	<0.001**
6001–12,000	730 (21.9%)	807 (17.9%)	162 (21.0%)	525 (24.2%)	
>12,000	586 (17.6%)	646 (14.4%)	114 (14.8%)	345 (15.9%)	
Associated chronic disease					
Yes	571 (17.1%)	764 (17.0%)	112 (14.5%)	430 (19.8%)	0.003**
No	2768 (82.9%)	3734 (83.0%)	660 (85.5%)	1742 (80.2%)	
Family history of cancer					
Yes	304 (09.1%)	504 (11.2%)	60 (07.8%)	197 (09.1%)	0.001**
No/do not know	3035 (90.9%)	3994 (88.8%)	712 (92.2%)	1975 (90.9%)	

## Discussion

This study evaluated the general population's preference for CRC screening methods. It is a representative study of Saudi Arabia due to the high number of responses and their equal distribution throughout the country. Studies suggest that patient choices for CRC screening modalities vary by country [20-21]. In our study, stool FIT was the most preferred screening modality by the general population (41.7%), while stool fecal occult blood testing (31%) and colonoscopy were the second and third options. Several papers indicated colonoscopy as the most preferred choice of screening modality for CRC [14-15,22-24]. Wong et al. [25] further indicated that the screening method using colonoscopy could be a better choice due to its ability to eliminate the adenomatous polyp. However, some papers suggested that FIT is the more effective CRC screening method [21,26], which was consistent with our findings.

The FIT screening method was more prevalent in the younger age group, females, unemployed respondents, and those who had a low-income level, while colonoscopy was more preferred by those who had associated chronic diseases. The preference of low-income residents for the FIT supports its usefulness for easing the economic burden. This is in contrast with the paper of Cho et al. [14]. They reported that FIT was more preferred by elderly patients, while colonoscopy was preferred by patients with higher education levels, a higher income level, or individuals with a family member or acquaintance with a history of CRC. On the other hand, we noted that the preference for the CRC screening method based on nationality and educational level did not vary significantly among the groups, which was in accordance with the paper of Al-Masoudi et al [15]. Although our study established FIT as the most suitable (33.5%) and preferable (41.7%) CRC screening method, the overall concern of respondents when choosing a screening modality is about how the test will be performed (50.4%) and the accuracy of the screening method (46.9%). As Tfaily et al. [1] emphasized, awareness about CRC screening is important to ease down the stigma of shame and embarrassment that attests to being the major stumbling block toward the willingness to undergo a CRC screening test.

The surgical field has witnessed fast and ongoing technological advancements in recent years. Adopting the IoT concept in surgical practice was one of the most revolutionary developments. Less time is spent doing surgery, more people have access to high-quality care, and surgical education is safer and more efficient. These are the key tangible benefits of IoT integration [27]. Thus, to add to this field through our study, we modified the application of FIT in the country and provided a home take-up, delivery of the sample test to the hospital, and the reach of its results through a phone application. With this addition, we eased access to the screening test at a low cost, which benefits all socioeconomic levels in the country. In low-income, uninsured populations, Van der Steen compared the advantages and effectiveness of the fecal immunochemical test to the colonoscopy. It turned out that the FIT prevented more CRC deaths than the colonoscopy, which only screened those who could afford it and thus only resembled a small portion of the targeted population [28]. The highest responses we got in our study were from the young age group, and that can be explained as they represent the largest number in the population of Saudi Arabia [19]. Most of them preferred FIT as a screening method for CRC, which supports the initiation of a national colorectal cancer screening program that suits the preferences of the population and meets the requirements of public and private healthcare centers in Saudi Arabia.

Respondents preferred FIT due to its accuracy (47.9%) or being upset with other screening modalities (40%). Others preferred fecal occult blood tests due to the duration of the screening (41.7%) and preparation requirements before the screening (41.2%). These findings are almost consistent with the paper of Calderwood et al. [22]. Based on their accounts, patients who preferred colonoscopy chose accuracy (76%) and frequency of testing (10%), whereas patients who preferred a stool-based test chose discomfort (52%) and complications (23%) as the most important features. In another published study done in the United States [11], they documented that the majority are more than willing to undergo a colorectal cancer screening test if the test does not involve radiation (73%), does not involve the insertion of a tube or device into the rectum (78%), does not involve a pre-procedural bowel cleansing regimen (73%), and does not involve sedation (60%). However, in Korea [14], researchers indicated that 12.9% of patients who underwent screening tests had bad experiences with both FIT and colonoscopy. Despite some barriers to undergoing CRC screening, the importance of taking the test is vital. As Hyams et al. [24] suggested, the effectiveness of the screening method is the most important criterion for making a decision.

Moreover, most of the respondents (92.8%) did not discuss the early colorectal cancer screening methods with their doctors for CRC, and only 2.8% were able to do so. Likewise, 93.4% of the respondents have not tried the CRC screening. Perhaps this is because the majority of our participants were in the younger age group (18-25 years) and have not reached the required age bracket for CRC screening practices. According to publications, patients are more than willing to undergo CRC screening [2,11], but the actual screening test did not reach the required target [1,2].

The risk factor for CRC is important to tackle since it could be associated with the willingness to undergo CRC screening. In our study, physical inactivity (23.6%), smoking (10.4%), and eating red meat (8.1%) were the most commonly mentioned risk factors for CRC. Eating red meat, a low-fiber diet, and a low intake of vegetables and fruits were determined as the most common risk factors for CRC, which were reported in Korea [14], Spain [2], and Lebanon [1].

Limitations

First, an unavoidable selection bias existed in our web-based survey because the study only involved participants who had access to the internet. Second, the results are based on a single survey of the population of Saudi Arabia. Therefore, the generalization of the results of this study to other countries is limited. Finally, the researchers developed the survey because there is no previous well-established, standardized questionnaire covering this topic to the best of our knowledge.

## Conclusions

Stool fecal immunochemical testing was preferred by the general population more than fecal occult blood tests or colonoscopies. This preference is likely demonstrated by females who are younger, unemployed, have a low monthly income, and have a family history of cancer. This research could represent a database that helps with the initiation of a colorectal cancer screening program that suits the preferences of the general population of Saudi Arabia. Moreover, awareness campaigns are necessary to increase the willingness of the general population to undergo colorectal cancer screening, specifically after the age of 45. Finally, early screening and detection are necessary to reduce the burden and costly treatment of any disease, including CRC.

## Appendices

Informed consent

We are a research group from Imam Mohammed Ibn Saud Islamic University - College of Medicine. We like to invite you to participate in our survey which is targeting above 18 years old Saudi citizens and residents who are free of active colorectal cancer.

We would like you to answer questions regarding your demographic data, medical history, and your preference of the colorectal cancer screening method.

If you agree to participate please know that the survey will not take more than 5 minutes and all answers will be handled with privacy and discreteness and will be only accessible by the research team only. Also, your participation is not mandatory and you have the right to not participate or quit anytime you want.

Thank you for your time and your cooperation.

Main Author: Dr. Mohammad Bukhetan AlHarbi Email: .sa

Do you agree to participate? ·      Yes ·      No

Background information

Age 18-25 26-40 41-50 51-60 61-70 71 and above 

Gender Female Male

Living at Eastern region Western region Northern region Southern region Middle region

Nationality Saudi Resident in Saudi Arabia

Highest level of education

Primary high school Bachelor degree Master degree, PhD degree and above Health practitioner 

Current employment. Employed full-time Employed part-time Self-employed Unemployed

Income per month in Saudi riyal. Less than 6000 6000-12,000 12,000-25,000 More than 25,000

Do you have any chronic diseases (you may check more than one)? No Inflammatory bowel disease (Crohns disease, ulcerative colitis) Diabetes mellitus Hypertension Obesity Other: __________________________________________

Which of the following applies (you may check more than one)? History of colorectal polyp Not physically active Diet rich in red meats Smoking Alcohol use None of the above 

Personal history of colorectal cancer Previous history of colorectal cancer (now free of cancer) I have colorectal cancer No previous history of colorectal cancer

Family history of cancer Colorectal cancer Other... No cancer\Do not know

Has your doctor ever discussed colon cancer screening with you? Yes No Do not remember

Have you ever had any of the following tests done in the past (you may check more than one)? No I Have not Fecal stool occult blood; stool samples provided in a container to check for blood Stool fecal immunochemical test; stool sample taken by a brush to check presence of blood by antibodies Sigmoidoscopy; flexible tube inserted up to the sigmoid colon Colonoscopy; flexible tube inserted which examines the large bowel and distal part of the small bowel Colorectal consultation

Description of screening tests

Based on the following information, which would suit you more? 

How frequently each test needs to be performed:

**Table 4:** How frequently each test needs to be performed?

Fecal stool occult blood	Performed at home, should be repeated every year
Stool fecal immunochemical test	Should be repeated every 3 years, automatic delivery of the result to your doctor
Sigmoidoscopy	Performed at clinic/hospital, repeated every 3 years
Colonoscopy	Performed at clinic/hospital, should be repeated every 5 years

Fecal stool occult blood Stool fecal immunochemical test Sigmoidoscopy Colonoscopy

Based on the following information, which would suit you more? 

**Table 5:** How is each test performed?

Fecal stool occult blood	You collect a stool sample from each of two or three bowel movements in a clean container, usually taken on consecutive days, and then use an applicator stick to apply a smear of stool to a specific area of a card. It takes about 10 minutes.
Stool fecal immunochemical test	At home, you use a stick or brush to obtain a small amount of stool. Automatic delivery of the result to your doctor. The fecal immunochemical test (FIT) uses antibodies to detect blood in the stool. It takes about 10 minutes.
Sigmoidoscopy	For this test, the doctor puts a short, thin, flexible, lighted tube into your sigmoid colon. A sigmoidoscopy usually takes 15 to 20 minutes.
Colonoscopy	For this test, the doctor puts a short, thin, flexible, lighted tube into your rectum. A colonoscopy typically takes about 30 to 60 minutes.

Fecal stool occult blood Stool fecal immunochemical test Sigmoidoscopy Colonoscopy

Based on the following information, which would suit you more? 

**Table 6:** Discomfort from each test.

Fecal stool occult blood	None, you need to visit the doctor the interpret the result
Stool fecal immunochemical test	None, no need to visit the doctor
Sigmoidoscopy	You may feel a strong urge to have a bowel movement when the tube is inserted. You may also have brief muscle spasms or lower belly pain during the test.
Colonoscopy	Abdominal discomfort or pain caused by cramping or bloating.

Fecal stool occult blood Stool fecal immunochemical test Sigmoidoscopy Colonoscopy

Based on the following information, which would suit you more? 
 

**Table 7:** Possible complications associated with each test.

Fecal stool occult blood	None
Stool fecal immunochemical test	None
Sigmoidoscopy	Bleeding and perforation
Colonoscopy	Adverse reaction to the sedative used during the exam, bleeding and perforation

Fecal stool occult blood Stool fecal immunochemical test Sigmoidoscopy Colonoscopy

Based on the following information, which would suit you more?
 

**Table 8:** Preparation before each test is performed.

Fecal stool occult blood	None
Stool fecal immunochemical test	None
Sigmoidoscopy	You need to empty your colon, which can be done by following a clear liquid diet the day before, take a laxative the night before the exam, and use an enema kit.
Colonoscopy	You need to empty your colon, which can be done by following a clear liquid diet the day before, take a laxative the night before the exam, and use an enema kit. Must come with an escort because sedation is given.

Fecal stool occult blood Stool fecal immunochemical test Sigmoidoscopy Colonoscopy Based on the following information, which would suit you more?
 

**Table 9:** The accuracy of each test.

Fecal stool occult blood	FOBTs only detect approximately 79% of colorectal cancer and polyps
Stool fecal immunochemical test	FIT detects 95% of colon cancers and 90% of large colorectal polyps
Sigmoidoscopy	Sigmoidoscopy can detect only 70% of cancers and polyps
Colonoscopy	The overall accuracy of colonoscopy was 94%

Fecal stool occult blood Stool fecal immunochemical test Sigmoidoscopy Colonoscopy

Based on the following information, which would suit you more?

**Table 10:** What would be done if each test is abnormal?

Fecal stool occult blood	You need additional testing to locate the source of the bleeding
Stool fecal immunochemical test	You need additional testing to locate the source of the bleeding.
Sigmoidoscopy	Depending on the findings, you may need additional testing such as a colonoscopy so that any abnormalities can be examined more thoroughly, biopsied or removed.
Colonoscopy	Diagnosis is made with this test. No further testing is needed.

Fecal stool occult blood Stool fecal immunochemical test Sigmoidoscopy Colonoscopy

Review

With the help of the information you have read, which testing type would you choose if given a choice by your doctor?

**Table 11:** Review.

Fecal stool occult blood	Frequency: Should be repeated every year. Performed by: stool sample from each of two or three bowel movements. It takes about 10 minutes. Discomfort: None. Complications: None. Preparation: None. Accuracy: FOBTs only detect approximately 79% colorectal cancer and polyps. If the test is abnormal: You need additional testing to locate the source of the bleeding.
Stool fecal immunochemical test	Frequency: Should be repeated every 3 years. Performed by: At home, you use a stick or brush to obtain a small amount of stool. It takes about 10 minutes. Discomfort: None. Complications: None. Preparation: None. Accuracy: FIT detects 95% of colon cancers and 90% of large colorectal polyps. If the test is abnormal: You need additional testing to locate the source of the bleeding.
Sigmoidoscopy	Frequency: should be repeated every 3 years. Performed by: the doctor puts a short, thin, flexible, lighted tube into your rectum. A sigmoidoscopy usually takes 15 to 20 minutes. Discomfort: urge to have a bowel movement when the tube is inserted. Brief muscle spasms or lower belly pain during the test. Complications: Bleeding and perforation. Preparation: You need to empty your colon. Accuracy: Sigmoidoscopy can detect only 70% of cancers and polyps. If the test is abnormal: Depending on the findings, you may need additional testing such as a colonoscopy.
Colonoscopy	Frequency: Should be repeated every 5 years. Performed by: For this test, the doctor puts a short, thin, flexible, lighted tube into your rectum. A colonoscopy typically takes about 30 to 60 minutes. Discomfort: abdominal discomfort or pain caused by cramping or bloating. Complications: Adverse reaction to the sedative used during the exam, Bleeding and perforation. Preparation: You need to empty your colon, must come with an escort because sedation is given. Accuracy: The overall accuracy of colonoscopy was 94% If the test is abnormal: Diagnosis is made with this test.

Fecal stool occult blood Stool fecal immunochemical test Sigmoidoscopy Colonoscopy

Please select the three most important qualities of a test from the list below that influenced your decision on choosing a particular test

Frequency of the test How the test is Performed Discomfort from the test Complications of the test Preparations for the test Accuracy of the test What to do If the test is abnormal
 
